# Rapid Rehabilitation Program Can Promote the Recovery of Gastrointestinal Function, Speed Up the Postoperative Rehabilitation Process, and Reduce the Incidence of Complications in Patients Undergoing Radical Gastrectomy

**DOI:** 10.1155/2022/1386382

**Published:** 2022-03-25

**Authors:** Xi Wang, Yan Li, Wei Yang

**Affiliations:** ^1^Department of Gynecology, China-Japan Union Hospital of Jilin University, Changchun, China; ^2^Department of Nursing, China-Japan Union Hospital of Jilin University, Changchun, China; ^3^Department of Hepatopancreatobiliary Surgery, China-Japan Union Hospital of Jilin University, Changchun, China

## Abstract

**Objective:**

To explore the influence of rapid rehabilitation programs on gastrointestinal function, rehabilitation process, and complications of patients undergoing radical gastrectomy.

**Methods:**

Of ninety-eight radical gastrectomy cases assessed for eligibility from January 2018 to July 2020, 43 patients who received routine perioperative nursing were assigned to the control group (CG), and 55 patients given a rapid rehabilitation program were assigned to the research group (RG). The recovery of gastrointestinal function, pain, nutritional status, complications, rehabilitation process, quality of life, and nursing satisfaction were compared.

**Results:**

After nursing, in contrast to the CG, the RG showed significantly better recovery of gastrointestinal function (the first time to eat (*t* = 7.701, *P* < 0.01), the first time to anal exhaust (*t* = 9.342, *P* < 0.01), the first time to defecation (*t* = 2.061, *P*=0.040), and the recovery time to bowel sounds (*t* = 16.030, *P* < 0.01)), notably improved pain and nutritional status, and showed fewer complications (*X*^2^ = 9.385, *P*=0.002). Rapid rehabilitation protocol also showed shorter recovery time and higher quality of life and nursing satisfaction of patients versus the routine perioperative nursing (all *P* < 0.05).

**Conclusion:**

The rapid rehabilitation program can accelerate the recovery of gastrointestinal function and postoperative rehabilitation and reduce the incidence of complications in patients undergoing radical gastrectomy.

## 1. Introduction

Gastric cancer (GC) is the most frequent malignancy of the digestive tract, ranking the third in causes of carcinoma death in the world [1]. In China, GC cases account for more than 40% of all the new cases in the world and 25% of all malignancy deaths [2, 3]. The number of new GC cases has reached over 1 million annually worldwide [4]. Bad dietary habits are the main culprit for the development of GC [5]. Radical gastrectomy, radiotherapy, and chemotherapy are the mainstay for the treatment of GC. Patients with early GC are well managed after radical gastrectomy and postoperative chemotherapy, with a postoperative 5-year survival rate reaching 90% [6]. Perioperative high-quality nursing can accelerate recovery and improve the prognosis of GC patients.

With the rapid development of the economy and medical quality, patients and their families have higher requirements for clinical nursing quality, which is highly associated with nursing satisfaction [7]. There exists an urgent need to employ high-quality nursing for postoperative recovery [8, 9]. The rapid rehabilitation program is developed to strengthen rehabilitation procedures and accelerate the postoperative recovery of patients [10]. At present, rapid rehabilitation has been widely applied in the clinic. Pagnotta et al. [11] suggested that, for patients undergoing unilateral total knee arthroplasty, rapid rehabilitation can significantly shorten the hospital stay of patients. However, there are few clinical studies on its application in radical gastrectomy, and the influence of this nursing mode on patients' rehabilitation and the complication rate is unclear.

Therefore, this study used rapid rehabilitation for patients undergoing radical gastrectomy to explore its application value in GC patients and provide a reference for clinical practice.

## 2. Materials and Methods

### 2.1. Clinical Data

Ninety-eight radical gastrectomy cases from January 2018 to July 2020 were identified as the research participants, of which 43 patients who received routine perioperative nursing were enrolled into the control group (CG) and 55 patients who received a rapid rehabilitation program were enrolled into the research group (RG). All patients were diagnosed with gastric carcinoma (GC) and met the diagnostic criteria as per the 2016 ESMO diagnostic guidelines [12]. All the eligible patients had complete clinical data, a possible survival time of over 3 months, and the indications of radical gastrectomy, and patients and their families provided written informed consent. The patients who dropped out halfway or the patients with the inadequate function of vital organs, communication barriers, and poor compliance were excluded. This study was approved by the Ethics Committee of our hospital.

### 2.2. Nursing Methods

Patients in the CG were given routine nursing during the perioperative period. Preoperative: the diet instruction, the related knowledge, and precautions of diseases and operations were informed to patients. Intraoperative: the nursing staff strictly followed aseptic operations and actively cooperated with the doctors. Postoperative: the changes of vital signs were strictly monitored, and the patients were given medication and dietary instruction.

Patients in the RG received a rapid rehabilitation program. Preoperative: the nursing staff explained disease-related knowledge and treatment methods to patients and their families in detail, positively communicated with patients, and answered their questions to relieve their negative emotions. The nutritional status of patients was evaluated, individualized dietary guidance was given to patients, and enteral nutrition support was performed when necessary. Patients were fasted and abstained from water 6 hours before operation, and 500 ml of 10% glucose solution was given orally 2 hours before operation. Intraoperative: the operating room temperature was maintained between 23°C and 26°C, and the infusion was heated to about 37°C before injection for the prevention of hypothermia. The temperature of the peritoneal lavage fluid was kept at 39°C. When the vital signs of patients were stable, the anastomosis instruments and abdominal drainage tube were withdrawn. The nursing staff should follow the doctor's advice to apply antihypertensive drugs and spectrum antibiotics to maintain stable blood pressure and prevent infection. Postoperative: the nursing staff should monitor the changes in the vital signs of patients and regularly adjust a comfortable position for patients. The patients were informed that there will be pain after operation, and the patients could be distracted by listening to music and acupuncture after using the analgesia pump to relieve the pain of the patients. In severe cases, oral analgesics were applied. The wound healing of patients was observed regularly, contaminated dressing needles were replaced in time, and catheters were cleaned regularly. After the first postoperative exhaustion, they were allowed to eat a small amount of liquid food. After the vital signs were stable and the pain was relieved, the patients were encouraged and instructed to perform on-bed exercise and rehabilitation training to shorten the rehabilitation time.

### 2.3. Outcome Measures

The main outcome measures were as follows: the postoperative recovery of gastrointestinal function was observed. The pain of the two groups before and after nursing was tested by the visual analogue scale (VAS) score. A higher score indicates more severe pain. The occurrence of postoperative complications and the rehabilitation process of the two groups were monitored.

The secondary outcome measures were as follows: SGA nutrition evaluation scale [13] was applied for evaluating the nutritional status of the CG and the RG after nursing, and the evaluation criteria are shown in Table 1; SF-36 scale was applied for evaluating the quality of life (QOL) of the CG and the RG before and after nursing, with a full score of 100 points, and a higher score indicates higher QOL. The self-made Nursing Satisfaction Questionnaire was applied for evaluating patients' nursing satisfaction [satisfaction = (satisfied + basically satisfied)/total cases × 100%].

### 2.4. Statistical Analysis

SPSS 26.0 was applied to statistically analyze the collected data, and GraphPad Prism 8 was used to visualize the matching images. Counting data were represented as rate (%) and analyzed using the chi-square test, which was expressed by *χ*^2^. All measurement data (mean ± SD) were in the normal distribution. The independent sample *t*-test was applied for intergroup comparison and the paired *t*-test for intragroup comparison, all of which were expressed by *t*. Differences were considered statistically significant at *P* < 0.05.

## 3. Results

### 3.1. General Clinical Data

Comparison of the general clinical data revealed no statistical difference in age, sex, drinking history, body mass index (BMI), smoking history, dietary preference, ASA grade, tumor size, and TNM stage between the CG and the RG (*P* > 0.05, Table 2).

### 3.2. Postoperative Gastrointestinal Function Recovery

The recovery of gastrointestinal function in the two groups was statistically analyzed. It was found that the first time to eat, the first time to anal exhaust, the first time to defecation, and the recovery time to bowel sounds in the RG were notably shorter than those in the CG (*P* < 0.05, Table 3).

### 3.3. Comparison of Pain

**Figure 1:**
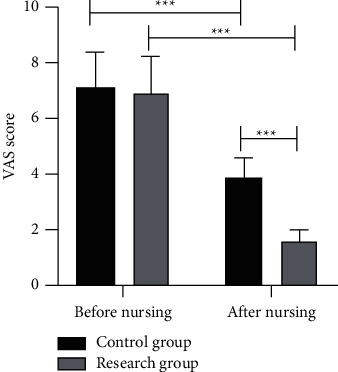
There was no significant difference in VAS scores between the CG and the RG before nursing. After nursing, the scores of the CG and the RG were lower than those before nursing, and the scores of the RG were significantly lower than those of the CG (^*∗∗∗*^*P* < 0.001).

The CG and the RG were similar in VAS scores before nursing (*P* > 0.05), but the scores of them after nursing decreased (*P* < 0.001), and the VAS scores of the RG were significantly lower than those of the CG (*P* < 0.001, Figure 1).

### 3.4. Comparison of Nutritional Status

The nutritional status of patients after nursing was evaluated by the SGA scale. The results revealed no difference between the CG and RG in the number of patients with grade B (*P* > 0.05), while the RG had more patients with grade A than the CG (*P* < 0.05), and fewer patients with grade C were observed in the RG than in the CG (*P* < 0.05), as shown in Table 4.

### 3.5. Occurrence of Complications

The incidence of postoperative complications in the RG was significantly lower than that in the CG (*P* < 0.05), as shown in Table 5.

### 3.6. Comparison of Rehabilitation

**Figure 2:**
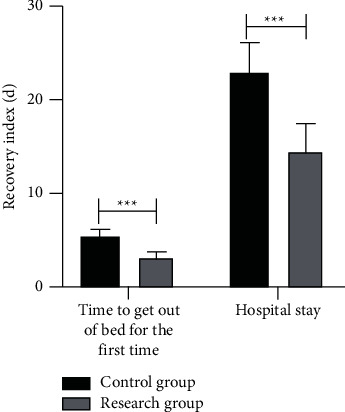
The time of getting out of bed for the first time and hospital stay in the RG were significantly shorter than those in the CG (^*∗∗∗*^*P* < 0.001).

The first time to get out of bed postoperatively and the hospital stay of the patients in the RG were significantly shorter than those of the CG (*P* < 0.001), as shown in Figure 2.

### 3.7. Comparison of the QOL

**Figure 3:**
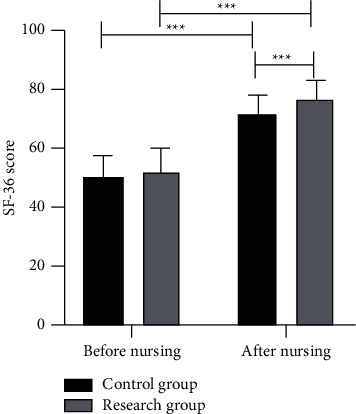
There was no significant difference in SF-36 scores between the CG and the RG before nursing. After nursing, SF-36 scores in both groups were significantly higher than those before nursing, and SF-36 scores in the RG were significantly higher than those in the CG (^*∗∗∗*^*P* < 0.001).

The SF-36 scale was applied to evaluate the QOL of the two groups before and after nursing. It was found that there was no significant difference in SF-36 scores before nursing (*P* > 0.05). After nursing, both groups showed significantly elevated QOL scores (*P* < 0.001), with better results observed in the RG versus the CG (*P* < 0.001), as shown in Figure 3.

### 3.8. Comparison of Nursing Satisfaction

After nursing, the satisfaction of patients with nursing work was counted. The results revealed that the nursing satisfaction of the RG was significantly higher than that of the CG (*P* < 0.05), as shown in Table 6.

## 4. Discussion

Radical gastrectomy is currently the standard therapy for locally advanced GC, and postoperative complications and surgical methods are the influencing factors for patients' long-term survival [14]. High-quality nursing is essential for improving the safety and prognosis of patients undergoing surgery during the perioperative period [15]. The application of perioperative high-quality nursing of lung carcinoma patients shows excellent efficiency and psychological benefits, enhances QOL, and reduces the occurrence of adverse events [16]. Therefore, the application of high-quality nursing in the perioperative period of surgery is of great significance to the rehabilitation of patients.

Rapid rehabilitation is a high-quality nursing mode developed in recent years, which mainly refers to the application of effective nursing measures during the perioperative period to promote patients' early recovery and shorten the hospital stay [17]. As the application of a rapid rehabilitation program in patients undergoing radical gastrectomy was marginally explored, this research explored the influence of this mode on gastrointestinal function and the rehabilitation process of patients undergoing radical gastrectomy. The results of the present study revealed a shorter time to perform daily activities in the RG than those in the CG, indicating that rapid rehabilitation could promote the recovery of gastrointestinal function after operation. Gastrointestinal dysfunction is a common postoperative complication, which prolongs the hospitalization time and increases nursing expenses and postoperative morbidity of patients, which severely compromises the rehabilitation of patients [18, 19]. The rapid rehabilitation adopted in this study has strong pertinence. The patients were given detailed dietary guidance or enteral nutrition and were encouraged to get out of bed earlier after surgery. SGA scale was applied to evaluate the nutritional status of patients after nursing. It was found that the RG had more patients with grade A and fewer grade C cases versus the CG, which indicated that rapid rehabilitation could significantly improve the nutritional status of patients after surgery. This may be attributed to the rapid rehabilitation of the gastrointestinal function of patients undergoing rapid rehabilitation programs, and combined with scientific dietary guidance and enteral nutrition support, the nutritional status of patients was significantly improved. Wang et al. [20] revealed that high-quality nursing for colorectal carcinoma patients undergoing laparoscopic examination can effectively reduce the incidence of postoperative complications, relieve the pain, and improve the gastrointestinal function of patients. In addition, the pain evaluation herein found no significant difference between the two groups before and after nursing, but significantly better alleviation of pain in the RG than that in the CG, which indicated that rapid rehabilitation could significantly reduce the postoperative pain of patients. In this research, physical methods such as acupuncture and listening to music were applied to divert patients' attention to relieve patients' pain. For patients with severe pain, oral analgesics were applied, and such a personalized analgesia method yields a significant effect on pain relief. Sharda et al. [21] indicated that measures such as listening to music during the perioperative period can effectively eliminate patients' anxiety and relieve their pain, which was similar to the results in the present study.

Surgical incision infection is one of the common postoperative complications and increases the disease and economic burden of patients [22]. Intestinal obstruction and postoperative bleeding are also common complications after radical gastrectomy [23], which can be effectively managed by high-quality nursing intervention. Here, the incidence of postoperative complications in the RG was significantly lower than that in the CG, indicating that rapid rehabilitation can significantly reduce the incidence of postoperative complications after radical gastrectomy for GC. Liu et al. [24] reported that high-quality nursing of patients during the perioperative period is beneficial to reduce the incidence of postoperative infection, reduce the pressure of operation, and improve the psychological state and prognosis of patients. Research by Xu et al. [25] also showed that rapid rehabilitation can effectively improve the psychological state of patients, reduce complications, relieve pain, promote postoperative rehabilitation, reduce economic pressure, and enhance the QOL. The first time of getting out of bed and the hospital stay of the patients in the RG were significantly shorter than those in the CG, and the QOL was significantly better than that in the CG, which indicated that the rapid rehabilitation had achieved better benefits in promoting postoperative rehabilitation and improving the QOL. Ding et al. [26] and Hu et al. [27] also indicated that the application of rapid rehabilitation in patients' perioperative period can promote the rehabilitation and enable the patients to obtain good surgical results. This is also similar to the results in the present study, and the benefits were attributable to the comprehensive and targeted nursing measures of a rapid rehabilitation program, which strengthens the patients' confidence in treatment, reduces the occurrence of complications, and shortens the hospital stay of patients. Finally, it was found that the nursing satisfaction of the RG was significantly higher than that of the CG, which confirmed that the rapid rehabilitation surgery nursing was recognized by the patients.

This study confirmed the high application value of rapid rehabilitation surgery nursing for patients undergoing radical gastrectomy in the perioperative period. The limitations of this study lie in the absence of the treatment compliance of patients, which will be evaluated to ensure the accuracy of the results.

To sum up, rapid rehabilitation surgical nursing is a safe and feasible nursing model to effectively promote the recovery of gastrointestinal function, reduce complications, speed up the process of postoperative rehabilitation, and improve the QOL, and it is worthy of clinical promotion.

## Figures and Tables

**Table 1:** SGA nutrition evaluation standard.

Index	Grade A (good)	Grade B (moderate)	Grade C (severe)
Recent weight change	None/elevated	Reduced by less than 5%	Reduced by more than 5%
Dietary changes	None	Declined	No food/low-calorie liquid food
Gastrointestinal symptoms	None/loss of appetite	Slight nausea and vomiting	Severe nausea and vomiting
Activity ability change	None/declined	Get out of bed and walk around	Bedridden
Stress response	None/low	Moderate	Severe
Muscle consumption	None	Mild	Severe
Thickness of the triceps skinfold (mm)	>8	6.5–8	<6.5
Ankle edema	None	Mild	Severe

**Table 2:** Comparison of general clinical data.

Factor	CG (*n* = 43)	RG (*n* = 55)	*t*/*χ*^2^	*P* value
Age (years)	51.23 ± 7.31	53.46 ± 8.24	1.396	0.166
Gender				
Man	19 (44.19)	28 (50.91)	0.437	0.509
Woman	24 (55.81)	27 (49.09)		
BMI (kg/m^2^)	21.84 ± 2.16	22.17 ± 2.13	0.756	0.451
History of smoking				
Yes	31 (72.09)	33 (60.00)	1.558	0.212
No	12 (27.91)	22 (40.00)		
History of alcoholism				
Yes	34 (79.07)	41 (74.55)	0.275	0.600
No	9 (20.93)	14 (25.45)		
Dietary preference				
Light	18 (41.86)	17 (30.91)	1.261	0.262
Greasy	25 (58.14)	38 (39.09)		
ASA grading				
I	23 (53.49)	25 (45.45)	0.623	0.430
II	20 (46.51)	30 (54.55)		
Tumor size				
<5 cm	33 (76.74)	39 (70.91)	0.422	0.516
≥5 cm	10 (23.26)	16 (29.09)		
TNM staging				
I ± II	35 (81.40)	40 (72.73)	1.010	0.315
III ± IV	8 (18.60)	15 (27.27)		
History of chemotherapy			1.558	0.212
Yes	31 (72.09)	33 (60.00)		
No	12 (27.91)	22 (40.00)		

**Table 3:** Recovery of gastrointestinal function after operation.

Group	CG (*n* = 43)	RG (*n* = 55)	*t*	*P*
The first time to eat after operation	0.38 ± 0.05	0.31 ± 0.04	7.701	<0.001
The first time to anal exhaust	3.05 ± 0.79	1.81 ± 0.52	9.342	<0.001
The first time to defecation	2.33 ± 1.39	1.84 ± 0.96	2.061	0.040
Recovery time to bowel sounds	1.53 ± 0.24	0.85 ± 0.18	16.03	<0.001

**Table 4:** Comparison of nutritional status.

Group	A	B	C
CG (*n* = 43)	7 (16.28)	15 (34.88)	21 (48.84)
RG (*n* = 55)	20 (36.36)	28 (50.91)	7 (12.73)
*χ* ^2^	4.877	3.757	15.42
*P*	0.027	0.053	<0.001

**Table 5:** Comparison of complications.

Group	Incision infection	Lung infection	Venous thrombosis of lower extremities	Ileus	Total
CG (*n* = 43)	5 (11.63)	2 (4.65)	3 (5.66)	2 (4.65)	30 (27.91)
RG (*n* = 55)	1 (1.82)	1 (1.82)	1 (1.82)	0 (0.00)	9 (5.45)
*χ* ^2^					9.385
*P* value					0.002

**Table 6:** Comparison of nursing satisfaction.

Group	Satisfied	Basically satisfied	Dissatisfied	Satisfaction
CG (*n* = 43)	14 (32.56)	18 (41.86)	11 (25.58)	32 (74.42)
RG (*n* = 55)	22 (40.00)	28 (50.91)	5 (9.09)	50 (90.91)
*χ* ^2^ value	—	—	—	4.804
*P* value	—	—	—	0.028

## Data Availability

The datasets used during the present study are available from the corresponding author upon reasonable request.
